# THE CAREGIVER IDENTITY IN CONTEXT: CONSEQUENCES OF IDENTITY THREAT FROM SIBLINGS

**DOI:** 10.1093/geroni/igac059.1169

**Published:** 2022-12-20

**Authors:** Marissa Rurka, J Jill Suitor, Megan Gilligan

**Affiliations:** University of Michigan - Center for Health and Research Transformation, Superior Charter Township, Michigan, United States; Purdue University, West Lafayette, Indiana, United States; Iowa State University, Ames, Iowa, United States

## Abstract

We draw from theories of identity and stress to examine the impact that siblings have on caregivers’ psychological well-being. Using data collected from 404 caregivers nested in 231 families as part of the Within-Family Differences Study, we conduct mediation analyses to examine whether perceived sibling criticisms are associated with caregivers’ depressive symptoms (a) directly and/or (b) indirectly through sibling tension. Qualitative data from the same caregivers give insight into the processes underlying statistical associations. We found an indirect relationship whereby perceived sibling criticisms were associated with greater sibling tension, which in turn was associated with higher depressive symptoms. Qualitative interviews show that efforts to mitigate the negative impact of sibling criticisms can lead to caregiver strategies that fuel sibling tension. These findings demonstrate how identity processes, as well as the family networks in which caregiving takes place, shape the experiences and consequences of parent care.

